# Double Trouble: Dengue Followed by COVID-19 Infection Acquired in Two Different Regions: A Doctor’s Case Report and Spatial Distribution of Cases in Presidente Prudente, São Paulo, Brazil

**DOI:** 10.3390/tropicalmed6030156

**Published:** 2021-08-25

**Authors:** Sérgio Munhoz Pereira, Charlene Troiani do Nascimento, Rodrigo Sala Ferro, Edilson Ferreira Flores, Elaine Aparecida Maldonado Bertacco, Elivelton da Silva Fonseca, Luiz Euribel Prestes-Carneiro

**Affiliations:** 1 Department of Emergency, Regional Hospital of Presidente Prudente, Oeste Paulista University, São Paulo 19050-920, Brazil; sergiopereira@unoeste.edu.br (S.M.P.); charlene@unoeste.edu.br (C.T.d.N.); rodrigoferro@unoeste.edu.br (R.S.F.); luiz@unoeste.br (L.E.P.-C.); 2 Statistics Department, School of Sciences and Technology, São Paulo State University, Presidente Prudente 19060-900, Brazil; efflores@fct.unesp.br; 3 Epidemiological Surveillance Service, Presidente Prudente 19010-081, Brazil; elaine.bertacco@unesp.br; 4 Institute of Geography, Santa Monica Campus, Federal University of Uberlandia, Uberlandia 38408-100, Brazil

**Keywords:** COVID-19, dengue, health care workers, clusters, neighborhoods

## Abstract

Co-epidemics of COVID-19 and dengue in dengue-endemic countries represent a serious public health concern. In Brazil, São Paulo state ranks first for cases and deaths from COVID-19, and dengue is endemic in most regions of the country. In 2020, an outbreak of dengue occurred in western São Paulo. We determined the spatiotemporal distribution of dengue in the context of COVID-19 cases in Presidente Prudente, a mid-sized city in western São Paulo. To illustrate the burden of both infections, a case report of a doctor and his family, infected with dengue and COVID-19, is presented. There were three clusters of dengue and COVID-19 in the periphery. A dengue cluster was found in a region where there were no corresponding COVID-19 cases. Meanwhile, there were COVID-19 clusters where dengue activity was lower. In 2020, the COVID-19 epidemic emerged when dengue reached its seasonal peak, resulting in a simultaneous outbreak of both diseases. Lower rates of dengue were found in the city compared with 2019, and the fear of patients with mild dengue symptoms about remaining in hospital and acquiring COVID-19 infection may be the main cause. Simultaneous spatial clusters of dengue and COVID-19 in environmentally and socioeconomically vulnerable areas can guide public health authorities in intensive interventions to improve clinical diagnosis, epidemiological surveillance, and management of both diseases. The patient and his family were first infected with dengue and he then carried COVID-19 to his family, reinforcing the risk of health care workers spreading the virus to the community. We highlight the epidemiological significance of presenting a case report and spatial analysis of COVID-19 in the same study in the context of a dengue outbreak.

## 1. Introduction

Co-epidemics of COVID-19 and dengue in dengue-endemic countries represent a serious public health concern. In Brazil, it is a dangerous combination for the public health system, which is already working above its capacity [1]. COVID-19 is caused by the SARS-CoV-2 coronavirus, and it has spread throughout the world at a speed and in a manner never seen before. Brazil ranks third for the number of patients infected and second for mortality rate worldwide [2]. As expected, São Paulo state, the most populous state of Brazil, and its capital, São Paulo, including the metropolitan area, ranks first for the number of cases and deaths from COVID-19 [3,4]. Individuals living in closed settings, including nursing homes and prisons, and those with an occupational health risk, particularly health care workers (HCWs), have increased vulnerability to the transmission of COVID-19. To date, several thousand HCWs globally have been reported to be infected [5].

Dengue is a mosquito-borne disease found in tropical and subtropical regions, mostly in urban and semi-urban settings [6]. According to the Brazilian Ministry of Health, in 2019, 1,544,987 probable cases were reported, with an incidence rate of 735.2 cases per 100,000 inhabitants, and the states of Minas Gerais, São Paulo, and Goias had 67.9% of the probable cases in the country [7]. By December 2020, 979,764 probable cases were reported, with an incidences rate of 466.2 cases per 100,000 inhabitants, and the Midwest region had the highest incidence [8]. The epidemiology of dengue and COVID-19 co-infections is complex, and many factors are responsible for driving their respective transmissions. Dengue and COVID-19 infections are difficult to distinguish due to their common clinical presentation, and both can be presented as a co-infection, leading to underdiagnosis [9]. This has devastating consequences in Latin American and Asian countries, where the dengue season coincided with the spread of COVID-19, and excess mortality and collapse of the health care system could not be attributed only to COVID-19 [10,11]. In the Americas, although the cumulative incidence rate in 2020 is lower than that reported in 2019, it is higher than the incidence rate reported for the 2016–2018 period. This situation occurred in parallel with the intense transmission of SARS-CoV-2 [11,12].

**Figure 1:**
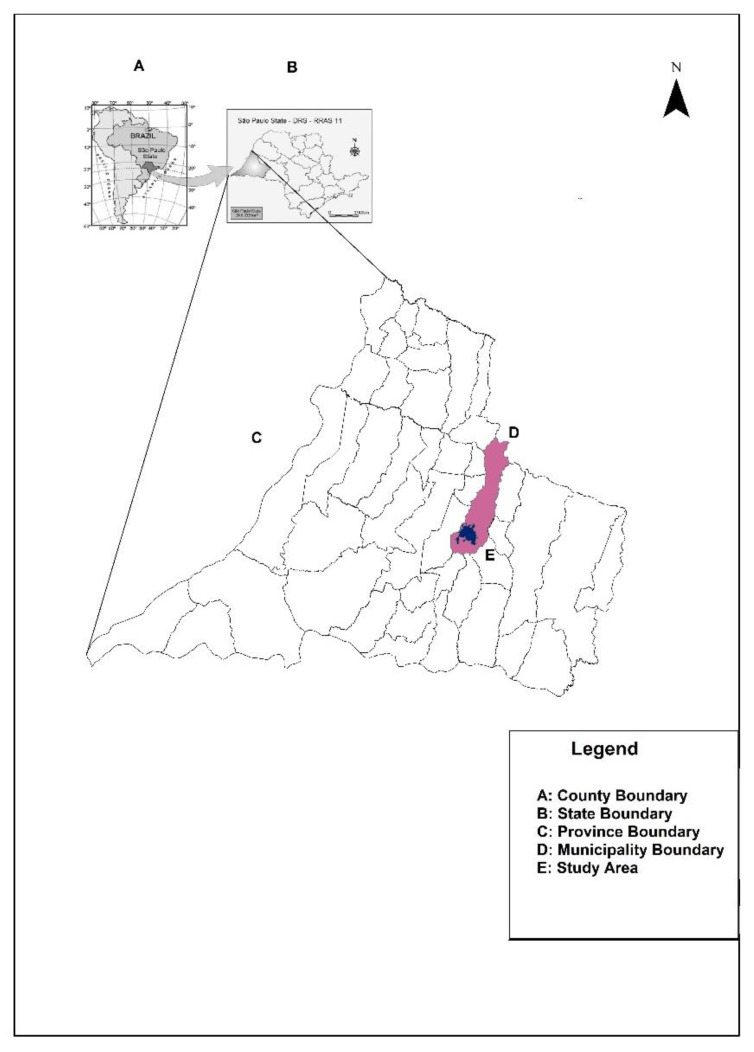
The study area adopted in this study: (**A**,**B**) the 26 states of Brazil and São Paulo state located in the southeastern region, highlighting the western region. (**C**) The 45 municipalities of the western region. (**D**) The municipality of Presidente Prudente and in (**E**) the urban area.

In São Paulo state, in 2019, a high incidence of dengue of between 100.01 and 8912.00 individuals per 100,000 inhabitants was registered in all regions [7]. Conversely, in 2020, a different landscape was observed, with low levels in other regions, and very high levels in the western region [8]. This region is located on the border of Paraná and Mato Grosso do Sul states, which are also dengue-endemic, with the same incidence levels. The reasons why rates of dengue are continuously increasing in the region are not well established. It is alarming that, in 2018 and in 2020, 56 and 58 irregular solid-waste deposits were found in the urban area along the river basins and in vacant household lots, respectively [13]. Incorrect disposal and storage of domestic waste by the population, increasing temperatures, deforestation [14], and *Aedes aegypti* mutations [15] are certainly environmental and socioeconomic factors that may contribute to the increase in the number of cases in Presidente Prudente, and they can be extended to the 45 municipalities of the western region (Figure 1A–C).

In 2020, in Presidente Prudente, the largest and most important city in the western region of São Paulo (Figure 1E), an outbreak of dengue was on course in the first months of the year at the same time as the COVID-19 pandemic was emerging. Based on our previous findings of vulnerable niches for dengue and visceral leishmaniasis [13], we hypothesized that, in these settings, a possible association of dengue in the context of COVID-19 cases was occurring. Identifying these areas would guide public health authorities in surveillance measures and improvements in health care infrastructure. Furthermore, dengue-infected people from an endemic region may spread the virus to other regions, and COVID-19 infected individuals may carry the virus from endemic areas to COVID-19-free areas. In April 2020, at the peak of the dengue outbreak and the emergence of the COVID-19 pandemic in Presidente Prudente, a doctor and his family living downtown, where low numbers of cases of dengue were expected, was diagnosed. A few days later, he was infected with COVID-19 in the metropolitan area of São Paulo, the epicenter of the disease in Brazil, and carried the virus to his family in the countryside. We highlight the epidemiological significance of presenting a case report and spatial analysis of COVID-19 in the same study in the context of a dengue outbreak. Furthermore, this study illustrates the burden of both infections in different settings and emphasizes the role of geoprocessing tools in monitoring the dispersion of both pathogens [3,4,16].

## 2. Settings, Materials, and Methods

The state of São Paulo is divided geographically into 15 mesoregions and 18 Regional Networks for Health Assistance (RNHAs). The western region harbors 45 municipalities and is administered by RNHA11, located in Presidente Prudente, the biggest and main city of mesoregion 8 [17]. The city is a mid-sized urban center about 560 km from the state capital, São Paulo. According to the census by the Instituto Brasileiro de Geografia e Estatística (IBGE) [18], in 2020, the estimated population was 230,371 inhabitants. (Figure 1D,E).

Kernel density was used as a method to show hotspots of dengue and COVID-19 in Presidente Prudente. Kernel density calculates the density of point features, creating a raster output grid with the intensity of a given phenomenon. The method uses a smoothly curved surface over each occurrence point, presenting higher values at the geolocation and diminishing in the opposite direction. This study used a pixel size output of 100 m. The density of each output raster cell (pixels) was calculated by adding the values of all surfaces in the kernel where they overlap the center of the raster cell. An Excel spreadsheet containing the addresses (places of residence) of positive cases of dengue and COVID-19 in the study period in the urban area of Presidente Prudente was provided. This file was exported to GIS ArcGIS (ArcGIS 10-7-1 software) and the column of addresses was geocoded, generating Points Shape, one for dengue and another for COVID-19 [19]. Unfortunately, we were unable to identify the location of the infection. Even with the individual’s address, it would be difficult to identify where the likely location of the infection occurred. Details of dengue and COVID-19 cases were obtained from the Department of Epidemiological Surveillance of Presidente Prudente, São Paulo state, Brazil.

## 3. Results

### 3.1. Case Report

A 61-year-old doctor lives with his family in Presidente Prudente in the countryside of São Paulo state, which is considered dengue-endemic. He remains in the city from Sunday night to Wednesday night, when he travels 560 km by bus to Osasco, a city in the metropolitan area of São Paulo’s capital. In Osasco, he remains from Thursday until Sunday afternoon, when he returns to Presidente Prudente. He works in a public tertiary reference hospital as an anesthesiologist for elective, general, vascular, orthopedics, and pediatric surgeries and emergency procedures such as stab wounds, firearm injuries, and open fractures. In Presidente Prudente, he takes care of his family and, on Wednesdays, he works as a teacher on a Medicine course. During this period, there were no in-person classes and he taught online. On Sunday the 26 of April, in Presidente Prudente, he presented fever (38.4 °C), arthralgia, diarrhea, headache, and abdominal pain, with dengue infection suspected. On 29 April, he presented diarrhea, myalgia, bitter taste in his mouth, lack of appetite, generalized exanthema and pruritus, and intense back pain. Due to an outbreak of dengue in Presidente Prudente, he was evaluated by an infectious disease specialist and dengue infection was suspected. The hemogram showed mild leukopenia (3636 cells/mm^3^; normal range, 4000–10,000/mm^3^) and thrombocytopenia with a platelet count of 117,000/mm^3^ (normal range, 150,000–400,000/mm^3^). On 29 April, Wednesday, he traveled from Presidente Prudente to Osasco, and on 30 April, 1 May, and 2 May, his dengue symptoms improved and ended on 3 May with normalized laboratory parameters (leukocytes, 6282 cells/mm^3^; platelets, 205,000/mm^3^). Dengue IgG antibodies were found on 28 May.

A few days after a complete recovery from dengue symptoms, on 13 May, Wednesday, he traveled from Presidente Prudente to Osasco and, on 14 May, he had mild back pain in the left hemithorax region. The next day, his back pain increased in intensity and was associated with mild dyspnea. Oxygen saturation on ambient air was 95–97%, with mild tachycardia (104 beats per minute). He was working in the Osasco hospital and was examined by an intensive care doctor, and COVID-19 infection was suspected. He underwent chest computed tomography (CT), which revealed ground-glass pulmonary opacities, sometimes associated with thickening of interlobular septa, with some confluent areas of consolidation, in multifocal, bilateral distribution, predominantly peripheral, and posterior, affecting up to 30% involvement of the lung parenchyma. Due to the clinical and tomography presentation, a real-time reverse transcriptase PCR (rRT-PCR) test for nasopharyngeal SARS-CoV-2 RNA was performed, with a positive result. He had medicated with azithromycin 500 mg (five days), levofloxacin 500 mg (seven days), methylprednisolone 40 mg once a day (five days), and dipyrone 500 mg every 6 h in the presence of pain or fever. On 15 May, he was put in quarantine and returned to Presidente Prudente. In the same week that he was diagnosed with COVID-19, seven doctors and two nurses at the same hospital were diagnosed and quarantined. On 24 May, he still had mild symptoms but, on 29 May, he was asymptomatic; COVID-19 IgG and IgM antibodies were found. As soon as there was clinical suspicion for COVID-19, he returned to Presidente Prudente; the patient, his son, and his wife remained in social isolation with symptomatic treatment and strict supervision of the clinical symptoms. In November 2020, his chest CT improved significantly, showing mild striation on the base of the left lung and mild dorsal spondylosis.

During the same period as his COVID-19 infection, his wife, aged 59 years, with no important comorbidities, had mild symptoms, including back pain and headache, and his son, aged 32 years with mild rhinitis and sporadic use of an anti-histamine, remained asymptomatic. An infectious diseases doctor was available, and the examinations for dengue and COVID-19 were prescribed and interpreted from our case report. COVID-19 IgM and IgG antibodies were found in both on day 14 after the appearance of symptoms. They were also infected by dengue with leukopenia and thrombocytopenia and positive IgG antibodies.

### 3.2. Dengue in the Context of COVID-19: Distribution of Cases of Dengue and COVID-19 Infection in the Urban Area of Presidente Prudente

**Figure 2:**
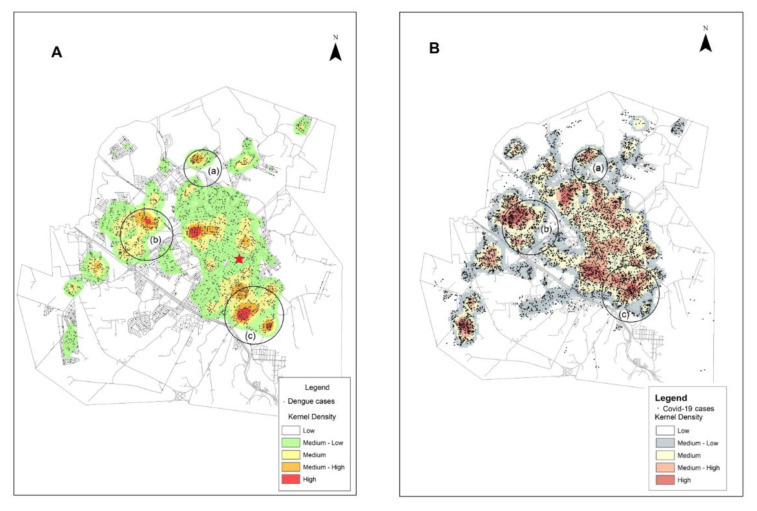
The spread of dengue (March 2019 to June 2020) (**A**) and COVID-19 (May to December 2020) (**B**) in the urban area of Presidente Prudente, São Paulo state, Brazil. One point per patient. The hotspots represent areas where a higher density of infected individuals was observed. The red star in (**A**) indicates the region where the patient and his family live. Circles (**a**–**c**) represent clusters of individuals infected with dengue and COVID-19.

In Presidente Prudente, outbreaks of dengue have occurred in the city in recent years. In 2019, an outbreak peaked between March and July, and in 2020 from February to June. In the subsequent months, cases still occurred but at much lower levels. The first individuals diagnosed with COVID-19 in Presidente Prudente was in April/May 2020 and, by December, 9164 individuals were notified (Figure 2). To understand the behavior of both viruses—endemic dengue and the COVID-19 pandemic—in the urban area of Presidente Prudente, and to detect spatial clusters for both viruses simultaneously, the spatial distribution of infected individuals was determined. Figure 2 shows that there were three clusters of patients infected with dengue and COVID-19 in different regions of the periphery. The neighborhoods of Cambuci, Vila Aurélio, José Rota, Jardim Paraíso, Santa Monica, and Itapura II are located on the east side (Figure 2a). The neighborhoods of Bela Vista, Santa Elisa, COHAB, São Geraldo, and Jardim São Paulo are on the west side (Figure 2b); a large area of Brasil Novo neighborhood is located on the north side (Figure 2c). In Figure 2A, there is a dengue cluster in the center of (Figure 2A) but there is no corresponding COVID-19 hotspot seen in (Figure 2B). Meanwhile, in Figure 2B, COVID-19 clusters appear between Figure 2a,b, and between Figure 2a,c, where dengue activity is lower. The patient in this case report lives downtown, with low levels of dengue-infected individuals (red star).

### 3.3. Dengue in the Context of COVID-19: Temporal Distribution of Cases of Dengue and COVID-19 in the Urban Area of Presidente Prudentein 2020

**Figure 3:**
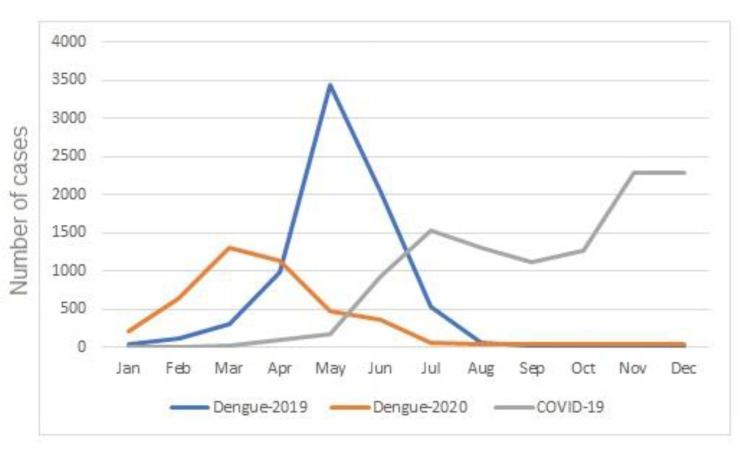
Dengue in the context of COVID-19. Temporal distribution of cases of dengue in 2019 and 2020 and COVID-19 in 2020 in the urban area of Presidente Prudente, São Paulo state, Brazil.

In Presidente Prudente, as well as in the 45 municipalities of the western region, the incidence of dengue gradually increases from the beginning of the year due to the rainy season and the high temperatures. In Presidente Prudente, in 2019, the outbreak peaked between March and July, and in 2020 from February to June. The COVID-19 epidemic emerged shortly before dengue reached its seasonal peak, resulting in a simultaneous outbreak of both conditions in the first months of 2020 (Figure 3).

## 4. Discussion

We showed that an outbreak of dengue was occurring in Presidente Prudente in the last few years, and in 2020, clusters of dengue and COVID-19 occurred mainly in regions of the periphery. Our case report is different from the dengue/COVID-19 infection reported previously in Brazil and worldwide. In Presidente Prudente, the doctor, his wife, and his son were infected by the dengue virus. In São Paulo, he was infected by COVID-19 at work and carried the virus to his family, highlighting the risk of HCWs spreading the virus to the community [20,21,22].

In contrast to our patient, in Reunion Island, France, an 18-year-old man was first infected with COVID-19 and, after recovery, he had symptoms of dengue [20]. COVID-19 and dengue co-infection were reported in Mayotte, another French island in the Indian Ocean [21]. In Buenos Aires, Argentina, a case report of a 25-year-old man diagnosed with COVID-19–dengue co-infection was reported [22]. Furthermore, in Buenos Aires, the clinical characteristics and outcomes of 13 hospitalized patients co-infected with dengue and COVID-19 were reported [23].

Working in a hospital in Osasco as an anesthesiologist, the patient was infected by COVID-19 in one of two probable ways: exposure to asymptomatic surgical patients who were subsequently found to have COVID-19, or exposure to an asymptomatic colleague in the doctors’ rest room.

HCWs are at a higher risk of being infected in their workplace [5,24]. In Brazil, data on HCWs infected by COVID-19 are scarce. According to the Brazilian Ministry of Health, up to 7 December 2020, 414,147 HCWs had been infected: nursing technicians and assistants (33.7%), nurses (15.1%), doctors (10.9%), and community health workers (5.2%) [25]. Up to 3 July 2021, 844 doctors working in the front line of COVID-19 had died and 77 (9.1%) were from São Paulo state [26]. In Brazil, although anecdotal notices about COVID-19–dengue co-infection are common, particularly in our region, few data have been published up until now. In July 2020, it was reported that a senator from Mato Grosso do Sul state, a COVID-19–dengue-endemic region, was hospitalized and diagnosed with a disease he called “COVENGUE”. One case of COVID-19 and dengue co-infection presented as a fatal stroke, was related in São José do Rio Preto, 273 km away from Presidente Prudente [27]. In Ceará state, in the northeast region, two cases of DENV-COVID-19 co-infection, potentially transmitted from a health care provider to family members residing in the same household, were reported [28].

Dengue, an environmentally related vector-borne viral disease, is one of the fastest spreading viral diseases, and it is endemic in over 100 countries, resulting in 40% of the world’s population living in an area at risk for dengue, according to the Pan American Health Organization [11]. Infected in Presidente Prudente by dengue, in the case presented here, the patient’s mild signs, symptoms, laboratory parameters, epidemiology, and clinical evolution, followed by IgM and IgG positive antibodies, confirmed the diagnosis. Note the reduction in the number of cases of dengue in 2020 compared with 2019 (Figure 3). A complex array of contributing factors may be involved, including the fear of patients with mild dengue symptoms about remaining in hospitals and acquiring COVID-19 infection; a considerable proportion of the population working at home, improving the control of vectors; and the efforts of the epidemiological surveillance department in the control of vectors and awareness of the population. To minimize overcrowding by people suspected of having dengue or COVID-19 infections in public health care centers, in May 2020, a specific public outpatient clinic was created only for patients suspected of having dengue, and laboratory tests were widely available in public and private health care systems. A similar reduction was found in the epidemic curve of dengue cases in Brazil in 2020 compared with 2019 [7,8]. This phenomenon has also been described in other environmentally related, dengue-endemic tropical countries. In India, in 2020, the burden of COVID-19 infection on decreasing rates of dengue became evident when comparing the epidemiological reports from the past 5 years. In Maharashtra, the third-largest state, in 2020, there was a marked decline in dengue cases of 84% compared with 2019 [29]. One of the main hypotheses was the depletion of health resources and the justified intense effort toward COVID-19, worsening the epidemiological profile of dengue in the country. In Sri Lanka, a reduction of 73.6% of confirmed cases of dengue between 2019 and 2020 was reported. Several factors may be triggered by these results, including weather conditions, the fear of hospitals due to the risk of hospital-acquired COVID-19 infection, and the closure of schools, offices, and airports during lockdowns [30].

In Presidente Prudente, when depicting the distribution of dengue and COVID-19, it is clear that both viruses were present in the whole urban area, from downtown to neighborhoods such as Brasil Novo in the north, Cambuci in the east, and Jardim São Paulo in the west. Simultaneous clusters of the two diseases were detected in neighborhoods of the east, west, and north regions with high population densities and low socioeconomic status, particularly in the east and north regions of the city. In the same areas, hotspots with a higher density of canine visceral leishmaniasis were recently identified [13]. These findings highlight these areas as priority targets for integrative interventions in health care services, education, sanitation, households, and economic development. Studies on co-infection of dengue and COVID-19 are limited to case reports and, as far as we know, no spatial analysis demonstrating clusters of infected individuals of both viruses simultaneously have been published. Recently, concomitant circulation of the COVID-19 pandemic and the triple arboviral epidemic of dengue, chikungunya, and Zika virus was demonstrated in various states of Brazil, highlighting the challenges faced by the Brazilian public health system in dealing with these viruses [4]. In a spatial analysis of the COVID-19 distribution pattern in São Paulo state, when the pandemic was recently introduced in Brazil, a higher density of cases was found in the metropolitan region of the capital São Paulo, including Osasco, where our case report is based [16]. In Figure 1, there is a dengue cluster in the center of (A), but there is no corresponding COVID-19 hotspot seen in (B). We suggest that the strong concentration of dengue cases in this region is related to the presence of environmental risk factors that favor the spread of vector-borne diseases. The area is bordered by the Limoeiro stream, with dense forest fragments and large non-urbanized areas with abandoned buildings, including slaughterhouses and tanneries. The area had a higher concentration of individuals infected with dengue in an outbreak in 2005. Furthermore, vectors of *Lu. longipalpis* and canine visceral leishmaniasis were first found in this setting [13]. In Figure 1B, COVID-19 clusters appear in regions where dengue activity is lower. These regions correspond to the downtown area, where the environmental conditions for disseminating dengue vectors are lower. We suggest that the presence of increased rates of dengue cases is linked mainly to environmental risk factors, and there is possibly no association with COVID-19 and environment. Our study adds regional and countrywide insights regarding dengue in the context of COVID-19 infection. They can be extended to countries sharing the same environmental (tropical climate, increasing temperatures, incorrect disposal and storage of domestic waste, lack of surveillance of vectors) and socioeconomic characteristics (poverty, low sanitation infrastructure, deficiency in health care services) as the western region of São Paulo state, and that are overwhelmed by mid-sized cities such as Presidente Prudente. There is a lack of population-based studies about dengue in the context of COVID-19 and, to the best of our knowledge, this is the first result in the field in Brazil.

Some shortcomings affect this study. The incidence of dengue has increased dramatically in recent decades in the western region of São Paulo state; however, most cases are asymptomatic and, hence, the numbers of cases of dengue are underreported, and many cases are misclassified. Our study population included only notified cases; asymptomatic cases were not captured. Similar to dengue, with COVID-19, a considerable number of patients are asymptomatic, and the number of cases reported is underestimated. In Presidente Prudente and countrywide, they may not reflect the real spread of the pandemic [31].

## 5. Conclusions

The detection of simultaneous spatial clusters of dengue and COVID-19 in environmentally and socio-economically vulnerable areas located in the periphery, in regions where dengue and visceral leishmaniasis were previously reported, can guide public health authorities in intensive interventions to improve clinical diagnosis, epidemiological surveillance, management, education, sanitation, and housing. In the case report, the patient and his family were first infected with dengue and, after this, he carried COVID-19 to his family; this fact highlights the risk of HCWs spreading the virus to the community. We highlight the epidemiological significance of presenting a case report and spatial analysis of COVID-19 in the same study in the context of a dengue outbreak.

## Data Availability

The database of dengue and COVID-19 patients identified by squares in the urban area of Presidente Prudente can be obtained at Epidemiological Surveillance of Presidente Prudente, Presidente Prudente, São Paulo state, Brazil.
